# Impact of family practice continuity of care on unplanned hospital use for people with serious mental illness

**DOI:** 10.1111/1475-6773.13211

**Published:** 2019-10-09

**Authors:** Jemimah Ride, Panagiotis Kasteridis, Nils Gutacker, Tim Doran, Nigel Rice, Hugh Gravelle, Tony Kendrick, Anne Mason, Maria Goddard, Najma Siddiqi, Simon Gilbody, Rachael Williams, Lauren Aylott, Ceri Dare, Rowena Jacobs

**Affiliations:** ^1^ Health Economics Unit Centre for Health Policy Melbourne School of Population and Global Health University of Melbourne Parkville Vic. Australia; ^2^ Centre for Health Economics University of York York UK; ^3^ Department of Health Sciences University of York York UK; ^4^ Department of Primary Care University of Southampton Southampton UK; ^5^ Hull York Medical School York UK; ^6^ Bradford District Care NHS Foundation Trust Bradford UK; ^7^ Mental Health and Addiction Research Group Department of Health Sciences University of York York UK; ^8^ Clinical Practice Research Datalink London UK; ^9^ Health Professions Education Unit Hull York Medical School York UK; ^10^ Service User York North Yorkshire UK

**Keywords:** continuity of care, family practice, hospital care, serious mental illness

## Abstract

**Objective:**

To investigate whether continuity of care in family practice reduces unplanned hospital use for people with serious mental illness (SMI).

**Data Sources:**

Linked administrative data on family practice and hospital utilization by people with SMI in England, 2007‐2014.

**Study Design:**

This observational cohort study used discrete‐time survival analysis to investigate the relationship between continuity of care in family practice and unplanned hospital use: emergency department (ED) presentations, and unplanned admissions for SMI and ambulatory care‐sensitive conditions (ACSC). The analysis distinguishes between relational continuity and management/ informational continuity (as captured by care plans) and accounts for unobserved confounding by examining deviation from long‐term averages.

**Data Collection/Extraction Methods:**

Individual‐level family practice administrative data linked to hospital administrative data.

**Principal Findings:**

Higher relational continuity was associated with 8‐11 percent lower risk of ED presentation and 23‐27 percent lower risk of ACSC admissions. Care plans were associated with 29 percent lower risk of ED presentation, 39 percent lower risk of SMI admissions, and 32 percent lower risk of ACSC admissions.

**Conclusions:**

Family practice continuity of care can reduce unplanned hospital use for physical and mental health of people with SMI.

## 1 INTRODUCTION

Serious mental illness (SMI) includes schizophrenia, schizoaffective disorder, bipolar disorder, and other psychoses. People with SMI have high rates of comorbidity,^1^ reduced quality of life,^2^ shortened life expectancy,^3^, ^4^ and high rates of emergency department (ED) presentations and unplanned hospital admissions.^5^, ^6^, ^7^ Finding ways to improve health care and outcomes for this group is therefore a high priority.^8^

Continuity of care is widely held to be beneficial for people with long‐term conditions, including SMI. It is valued by patients^9^, ^10^ and providers^11^ and considered good practice in mental health and family medicine,^12^, ^13^, ^14^ reducing fragmentation of care and facilitating better provider‐patient relationships.^15^ Relational continuity—the longitudinal relationship between a patient and a health care practitioner (or group of practitioners)^16^—is often the focus of efforts to improve continuity. To date, evidence has been mixed on whether relational continuity improves outcomes for people with SMI. Some studies have found that higher continuity is associated with lower mortality,^17^ reduced hospital admissions,^18^ and improved recovery from episodes of SMI,^19^ while others have found no association or even the reverse.^20^, ^21^, ^22^ Studies that have examined the relationship of continuity to costs have mostly found that higher continuity was associated with lower health care costs, although one showed an association with higher costs of community care^18^, ^23^, ^24^ It is important to clarify whether relational continuity is beneficial, since achieving higher continuity may increase costs and require trade‐offs with other elements of good care, such as flexibility to meet urgent care needs.^25^

Studies of relational continuity for people with SMI have most often considered visits within specialist mental health services, or across multiple types of service^21^ (which we term “across‐practice continuity”). However, in the UK family physicians provide much of the physical and mental health care for people with SMI and around a third of people with SMI are treated solely by their family physician.^26^ Policies such as named accountable practitioners have emphasized the importance of maintaining continuity with an individual family physician, not just a practice.^27^ The UK's National Health Service (NHS) provides publicly funded health care which requires patients to register with a specific family practice, so that patients face barriers to changing practices or attending different practices concurrently. In other health care systems, the role of family physicians in the care of people with SMI may be less prominent,^28^ and patients may be more likely to see physicians at different family practices, but initiatives such as the patient‐centered medical home in the United States have a similar focus on relational continuity with family physicians.^29^ Evidence is therefore needed on the impact of within‐practice family physician continuity on the physical and mental health of people with SMI, in addition to the existing literature on across‐practice continuity focused on specialist mental health care.

Continuity of care has other aspects beyond relational continuity, including informational and management continuity.^16^ In the United Kingdom, people usually register with a family practice and within that practice have a nominated physician who acts as a gatekeeper to and liaison with other health care services, including specialist mental health services. However, individuals can see any physician in that practice, especially for urgent appointments. Care plans for people with SMI document the patient's care needs, patterns of relapse, preferences for treatment, and social context^30^ and are stored with patient records and accessible by different practitioners seeing the patient. Care plans therefore promote informational continuity across family physicians in the same practice and may also promote management continuity, if the management approach is agreed and can be followed by all practitioners. A previous study showed that care plans for people with SMI were associated with a lower risk of unplanned hospital use, but that study did not account for relational continuity.^31^

Relational continuity is known to vary with observed individual characteristics such as age and sex,^32^, ^33^, ^34^ but continuity may also be influenced by factors that are usually unobserved, such as help‐seeking attitudes, disease severity, personality, or social context. If these unobserved factors also influence outcomes, the observed association between continuity and outcomes may be biased. For example, people who are more proactive in seeking care may receive higher continuity, but they may also have better outcomes because they seek care early or engage in preventive management. Conversely, family physicians may prioritize continuity for people with more severe illness, who nonetheless may have a higher risk of deterioration than those with less severe illness. To our knowledge, only one study has attempted to address unobserved confounding when examining the relationship between continuity of care and outcomes. It looked at the effect of relational continuity on emergency department attendance for people with diabetes and hypertension in Taiwan and measured continuity in 1 year and outcomes in the next.^35^ It employed an instrumental variable approach to account for confounding, with the relational continuity of family members of the patient as instrument. The results showed a stronger negative association between continuity and ED presentations with the instrumental variable approach than the standard approach.

We examined whether family physician relational continuity for people with SMI is associated with better outcomes, using the novel application of methods to account for time‐invariant unobserved confounding. The study objective was to investigate the hypothesis that continuity of care in family practice reduces unplanned hospital utilization.

## 2 METHODS

### 2.1 Study design

This observational cohort study used individual‐level family practice administrative data linked to hospital administrative data to investigate the relationship between family practice continuity of care for people with SMI and time to unplanned hospital use.

### 2.2 Sample

We used data from the Clinical Practice Research Datalink (CPRD), a database of anonymized patient records derived from over 600 family practices in England and broadly representative of the national population with respect to age and gender.^36^ The records were linked to Hospital Episode Statistics (HES), which capture all hospital admissions (for both physical and mental health) and ED presentations funded by the NHS. This covers the majority of these types of health care in England, since the NHS funds 88 percent of all health care expenditure^37^ and 92 percent of hospital care,^38^ and there are no privately funded emergency departments. The sample was all people with a diagnosis of SMI documented in primary care on or before March 31, 2014 (the end of the study period), whose records met CPRD quality standards, and who were registered during this period at a participating practice that met CPRD standards.^36^ Diagnoses of SMI were based on clinical information in routine practice data recorded in Read codes, an hierarchical coding system for clinical data that classifies diseases, patient characteristics, tests, and procedures^39^, ^40^ (see Table S1 for a list of the Read codes used in this study).

The start date of observation for each individual was the latest of: date of SMI diagnosis, date of registration at the practice plus 1 year of observation in primary care records, January 1 of the calendar year after the person turned 18, and April 1, 2007 (because data on ED presentations were only available from this date). The year of observation in primary care records allowed for observation of baseline characteristics as control variables. Additionally, the start date of observation for each individual was moved later if necessary so that no patients had an ED presentation or a hospital admission for at least 1 year prior to the start date, since hospital care could influence the level of continuity in primary care.

The observation period for each individual was divided into periods of 3 months dating from their first date of observation, with continuity measured in the prior 12 months. Individuals were followed until outcome or censoring, where censoring is due to the person changing family practice, death, or the end of the study period (March 31, 2014).

### 2.3 Outcome measures

We constructed three measures of unplanned hospital use from HES: (a) ED presentations, (b) unplanned admissions for SMI, and (c) unplanned admissions for ambulatory care‐sensitive conditions (ACSC),^41^ which are conditions thought to be particularly amenable to ambulatory care (such as diabetes, angina, cellulitis, and vaccine‐preventable diseases, but not SMI). Hospital admissions were classified using International Classification of Disease (ICD‐10) codes to identify SMI and ACSC admissions. (The codes used to classify ACSC admissions are listed in Table S2.) All ED presentations were included. For each type of outcome, we considered only the first observed instance (presentation or admission), since this could have influenced subsequent continuity.

The occurrence of the outcome is measured in the 3‐month period *t* and continuity is measured over a lookback period of the prior 12 months (4 × 3 month periods *t*−4 to *t*−1). That is, there is no overlap between the 12‐month period in which continuity is measured and the subsequent 3‐month period in which outcomes are observed.

The outcome variable is a binary variable for each 3‐month period indicating whether or not the event occurred in that period. For any individual who did not experience the outcome of interest (eg someone who did not present to ED during the period of observation), this variable is equal to zero for all periods. As we only analyzed time to first event, for any individual who did experience the outcome, the variable is equal to zero for all periods except the final period and equal to one for the final period, with all periods after the first event excluded from the analysis for that outcome.

### 2.4 Measures of relational continuity

We used three indices measuring different dimensions of family physician relational continuity.^42^ The Continuity of Care (COC) index measures dispersion of visits across family physicians within the patient's registered family practice, by capturing how many different practitioners are involved and how many visits occur to each. The Usual Provider of Care (UPC) index measures density of visits, being the proportion of a patient's visits that are with the family physician most frequently seen by the individual in that year out of the total number of visits at the practice. The Sequential Continuity (SECON) index measures the pattern of visits across different practitioners, using the proportion of consecutive pairs of visits which are to the same family physician out of the total number of consecutive pairs of visits at the practice. Each index ranges from zero (lowest continuity) to 1 (perfect continuity). Additional detail on each index is in the Tables S1‐S6, and illustrative examples are shown in Table 1.

**Table 1:** Examples of visit patterns and associated continuity of care indices

Scenario	Visit pattern	Number of visits	Number of practitioners	COC index	UPC index	SECON index
A	All visits with same practitioner	8	1	1	1	1
B	Each visit with a different practitioner	8	8	0	0.13	0
C	4 visits with one practitioner, then 4 with another	8	2	0.43	0.50	0.86
D	5 visits with one practitioner, then 3 with another	8	2	0.46	0.63	0.86
E	Alternating between 2 practitioners	8	2	0.43	0.50	0
F	As for scenario E but one extra visit with first practitioner	8	2	0.46	0.63	0.29

We measured continuity over 12 months (4 × 3 month periods), considering only face‐to‐face visits with family physicians. There is no standard level for “high” and “low” continuity, so we applied one recognized method that classified relational continuity as “high” if the level of continuity was above the median for the index, and “low” if at or below the median level.^43^, ^44^

A minimum of two visits is required to calculate COC and SECON, but to improve index stability we set the minimum to three visits. Periods with fewer than two visits in the prior 12‐month lookback period were included in the analysis with continuity categorized as “undefined.” We constructed a set of categorical variables based on visit frequency and whether continuity was low or high. This allowed for different effects of continuity for frequent and less frequent users of family practice, as suggested by previous research.^45^ Visit frequency was classified into low, moderate, and high: low (0‐2 visits), moderate (3‐5 visits), and high (6 or more visits). These categories correspond to tertiles of the full‐visit distribution: two visits is the 33rd percentile and five visits is the 66th percentile. Continuity indices were defined as low or high based on the median value of each index: COC low (0‐0.35), high (>0.35); UPC low (0‐0.67), high (>0.67); SECON low (0‐0.17), high (>0.17).

Periods were then classified into five categories according to continuity level and visit frequency in the prior 12 months: low visit frequency (with continuity undefined—the base category), moderate visit frequency with low continuity, moderate visit frequency with high continuity, high visit frequency with low continuity, and high visit frequency with high continuity.

### 2.5 Measure of informational/management continuity

This analysis captures management/ informational continuity separately from relational continuity according to whether the individual had a care plan documented by a family physician in the prior 12 months. Because we focus on within‐practice family physician continuity, we distinguish relational continuity from management and informational continuity represented by care plans. Doctors within a practice have access to the same medical records and may have similar approaches to management.^46^

### 2.6 Control variables

Individual characteristics measured at baseline were as follows: age, gender, ethnicity, deprivation of the person's neighborhood of residence,^47^ history of smoking, number of Charlson Index comorbidities,^48^ comorbid depression, diagnostic subgroup (schizophrenia and other psychoses, or bipolar disorder and affective psychoses) and number of years since diagnosis. Treatment for SMI was included as a time‐varying variable indicating that the individual had been prescribed an antipsychotic drug at least once in the 12‐month lookback period prior to the current period.

### 2.7 Statistical analysis

The necessity of creating periods for continuity measurement led us to employ discrete‐time survival analysis. Although the outcomes of interest are (effectively) continuous measures (since we have day‐level data on when these occur), these are converted into discrete outcomes for each period in order to match the measurement of continuity. The model evaluates the association between continuity in the prior 12 months and risk of the outcome in a particular 3‐month period.

A complementary log‐log (cloglog) proportional hazards model was fitted for each outcome. This model produces hazard ratios that are the discrete‐time equivalent of the Cox proportional hazards model used in a continuous‐time context.^49^ A flexible piece‐wise constant baseline hazard function was applied by specifying dummy variables for each 3‐month period. This assumes that the hazard function is constant within each period, but can vary across periods. The resulting exponentiated coefficients can be interpreted as hazard ratios, the discrete‐time counterpart of the hazard from a continuous‐time proportional hazards model.^49^ The hazard ratio is the proportional change in the underlying hazard of the outcome for a unit change in the variable.

The hazard rate (HR) at period *t* is the probability of observing the outcome for an individual in period *t*, conditional on the individual “surviving” in the sample to period *t* (ie, no censoring and the outcome was not observed in prior periods for that individual). The HR is a nonlinear function of time‐varying factors, time‐invariant factors, time‐period dummy variables representative of the baseline hazard, and normally distributed individual unobserved heterogeneity.

Our main modeling approach accounts for individual unobserved heterogeneity. Due to the incidental parameter problem^50^ of specifying individual fixed effects to represent such heterogeneity in nonlinear models, we instead assume unobserved heterogeneity is normally distributed and specify this as a linear function of the means of time‐varying variables. This is often termed a correlated random‐effects model, following Mundlak.^51^ The time‐varying variables were the care quality indicators plus the time‐varying covariate for antipsychotic treatment, while the remaining individual characteristics included as covariates were time‐invariant, captured at baseline. The variables representing the means of the time‐varying variables effectively capture confounding by unobserved time‐invariant individual factors (eg long‐standing illness, health‐seeking behavior) that drive both continuity and use of hospital services. The period‐specific levels of the time‐varying variables capture deviation from this long‐term average and can be interpreted as the effect of continuity specific to that 3‐month period, given the person's overall propensity to receive continuity of care. (See Tables S1‐S6 for more detail of the model.)

To allow comparison of our results to previous studies examining the effect of continuity of care, we also estimated models that did not specify individual heterogeneity as a function of the means of the time‐varying variables, the random‐effects model. These models allow for normally distributed individual heterogeneity but it is assumed to be uncorrelated with the explanatory variables contained in the model.

All models included observed individual characteristics as explanatory variables and adjusted standard errors for clustering at the practice level. We estimated separate models for each of the three continuity indices because of multicollinearity of the indices. All analyses were conducted using Stata v14.

### 2.8 Robustness checks

1. We tested the sensitivity of the results to the level of visit frequency at which continuity was classified as “undefined”. The minimum level for measuring continuity (and corresponding categories for low vs moderate visit frequency) was set to two or four visits rather than three visits as in our main analysis.
2. Given the significant physical health comorbidities of people with SMI, we examined an alternative to the two separate hospital admission outcomes: all‐cause hospital admissions, capturing all unplanned admissions for both physical and mental health conditions.
3. To investigate whether receiving specialist mental health care confounded the relationship between primary care quality and outcomes, we ran additional analyses capturing care in specialist mental health services. Data on specialist care from the Mental Health Services Minimum Dataset (MHMDS) were only available to link to the main dataset from April 1, 2011. We added a time‐varying variable to indicate whether the individual received any care in specialist mental health services in the prior 12 months and ran the analysis over the 3‐year period of observation to March 31, 2014.

## 3 RESULTS

### 3.1 Sample

The sample consisted of 19 324 individuals attending 215 practices, observed for 15.8 3‐month periods on average (range 1‐28 periods). Table 2 presents the characteristics of individuals in the sample. Half of the sample (50.3 percent) had an ED presentation at some point during the observation period, 13.1 percent had an admission for SMI, and 12.8 percent had an ACSC admission. Using a three‐visit minimum to define continuity, median (mean) values for each continuity index were as follows: COC 0.35 (0.46), UPC 0.67 (0.65), and SECON 0.17 (0.26). A care plan had been documented in the previous 12 months for 40 percent of the periods observed. The Spearman rank correlation between COC and UPC indices was 0.94 (*P* < .001), between COC and SECON was 0.55 (*P* < .001), and between UPC and SECON was 0.47 (*P* < .001). Mean COC in periods with a care plan in the previous 12 months was 0.47, compared with 0.45 in periods without a care plan in the previous 12 months (two‐sample *t* test of difference in means: *P* < .001); the equivalent for UPC was 0.67 vs 0.66 (*P* < .001) and for SECON was 0.24 vs 0.23 (*P* < .001).

**Table 2:** Sample characteristics (N = 19 324 individuals, 305 022 periods)

Characteristics fixed at baseline	N individuals (%)	
Age		
19‐35	5328 (27.6%)	
36‐45	4407 (22.8%)	
46‐55	3571 (18.5%)	
56‐65	2678 (13.9%)	
≥66	3340 (17.3%)	
Gender		
Female	9705 (50.2%)	
Male	9619 (49.8%)	
Index of multiple deprivation		
1 Least disadvantaged	3113 (16.1%)	
2	3546 (18.4%)	
3	3605 (18.7%)	
4	4484 (23.2%)	
5 Most disadvantaged	4576 (23.7%)	
Ethnicity		
Black and minority ethnicities	5609 (29.0%)	
White	13 715 (71.0%)	
Diagnosis category grouping		
Bipolar disorder and affective psychoses	6846 (35.4%)	
Schizophrenia and other psychoses	10 254 (53.1%)	
Both categories	2224 (11.5%)	
Years since diagnosis		
0‐1	5779 (29.9%)	
2‐5	3953 (20.5%)	
>5	9592 (49.6%)	
Number of Charlson comorbidities		
0	13 246 (68.5%)	
1	4726 (24.5%)	
2 or more	1352 (7.0%)	
History of depression		
No history of depression	8382 (43.4%)	
Comorbid depression	10 942 (56.6%)	
History of smoking		
Nonsmoker	5436 (28.1%)	
Current or ex‐smoker	13 888 (71.9%)	

During the observation period	N individuals (%)	Mean (SD)
Number of 3‐mo periods observed		15.8 (10.0)
At least one ED presentation	9719 (50.3%)	
At least one SMI admission	2525 (13.1%)	
At least one ACSC admission	2475 (12.8%)	

In each 12‐mo lookback period	N periods (%)	Mean (SD)
Low visit frequency (0‐2 visits)	125 513 (41.1%)	
Moderate visit frequency (3‐5 visits)	85 809 (28.1%)	
High visit frequency (6 or more visits)	93 700 (30.7%)	
Care plan	121 724 (39.9%)	
Antipsychotic medication	162 448 (53.3%)	
COC index		0.46 (0.32)
UPC index		0.65 (0.24)
SECON index		0.26 (0.30)

### 3.2 Association between continuity of care and unplanned hospital use

Table 3 presents the association between continuity of care and each outcome from the discrete‐time survival analyses, with relational continuity measured by the COC index. The results presented are HRs for the key variables of interest from our preferred model, the correlated random effects which accounts for unobserved confounding. Results are also presented for comparison from the model which does not account for unobserved confounding, the random‐effects model. (Full results for each outcome from the correlated random‐effects model are presented in Table S3.)

**Table 3:** Association of continuity measures with hazard of each outcome, and demonstrating the effect of accounting for confounding by time‐invariant unobserved characteristics

	Correlated random‐effects model^a^	Random‐effects model^b^
	Hazard ratio (95% CI)	Hazard ratio (95% CI)
ED presentation		
Relational continuity		
Moderate visit frequency (3‐5 visits)	0.89** (0.83‐0.96)	0.84*** (0.77‐0.91)
High COC index vs low COC index		
High visit frequency (6 or more visits)	0.92 (0.84‐1.00)	0.86*** (0.80‐0.92)
High COC index vs low COC index		
Information/management continuity		
Care plan vs none	0.71*** (0.66‐0.76)	0.94* (0.90‐0.99)
SMI admission		
Relational continuity		
Moderate visit frequency (3‐5 visits)	0.98 (0.82‐1.18)	0.98 (0.82‐1.16)
High COC index vs low COC index		
High visit frequency (6 or more visits)	0.90 (0.75‐1.08)	0.94 (0.82‐1.08)
High COC index vs low COC index		
Information/management continuity		
Care plan vs none	0.61*** (0.55‐0.68)	1.27*** (1.16‐1.40)
ACSC admission		
Relational continuity		
Moderate visit frequency (3‐5 visits)	0.77** (0.65‐0.91)	0.74*** (0.62‐0.88)
High COC index vs low COC index		
High visit frequency (6 or more visits)	0.73*** (0.62‐0.87)	0.71*** (0.61‐0.82)
High COC index vs low COC index		
Information/management continuity		
Care plan vs none	0.68*** (0.60‐0.77)	0.96 (0.87‐1.05)

Note

Continuity: low = ≤median COC index, high = >median COC index. Visit frequency: low = 0‐2, moderate = 3‐5, high = 6+ visits in 12 mo. Hazard ratios between two levels of continuity obtained as the ratio of exponentiated coefficients: HR_high/low_ = exp (*β*_high_)/exp (*β*_low_).

a This model accounts for confounding by unobserved time‐invariant individual characteristics, using the approach following Mundlak (1978).

b Random‐effects model assumes individual heterogeneity is uncorrelated with the explanatory variables.

* *P* < .05;

** *P* < .01;

*** *P* < .001.

Higher relational continuity as captured by the COC index was associated with 11 percent lower risk of ED presentation (HR 0.89, 95% CI 0.83‐0.96) for those with moderate visit frequency and 8 percent lower for frequent attenders but of borderline statistical significance (HR 0.92, 95% CI 0.84‐1.00, *P* = .057). Higher continuity was associated with 23 percent lower risk of ACSC admission (HR 0.77, 95% CI 0.65‐0.91) for those with moderate visit frequency and 27 percent lower for frequent attenders (HR 0.73, 95% CI 0.62‐0.87). Risk of SMI admission did not differ by level of continuity for moderate or frequent attenders.

Having a care plan documented in the previous 12 months was associated with 29 percent lower risk of ED presentation (HR 0.71, 95% CI 0.66‐0.76), 39 percent lower risk of SMI admission (HR 0.61, 95% CI 0.55‐0.68), and 32 percent lower risk of ACSC admission (HR 0.68, 95% CI 0.60‐0.77).

The standard approach (random effects) to modeling continuity, which does not account for unobserved confounding, produced different results, especially regarding care plans, as seen in the final column of Table 3. This approach found that higher relational continuity was associated with lower risk of ED presentation and lower risk of ACSC admission, at both moderate and high visit frequency, and that care plans were associated with higher rather than lower risk of SMI admission.

Table 4 shows that lower risk of ED presentations is stronger when relational continuity is measured with the SECON index than UPC or COC, and there was some association with lower risk of SMI admission with UPC and SECON, but otherwise the results have a similar pattern across the different indices.

**Table 4:** Association of relational continuity as measured by UPC index or SECON index with hazard of each outcome

	UPC index	SECON index
	Hazard ratio (95% CI)	Hazard ratio (95% CI)
ED presentation		
Relational continuity		
Moderate visit frequency (3‐5 visits)	0.90* (0.83‐0.98)	0.84*** (0.77‐0.92)
High continuity index vs low		
High visit frequency (6 or more visits)	0.97 (0.89‐1.05)	0.90** (0.84‐0.97)
High continuity index vs low		
SMI admission		
Relational continuity		
Moderate visit frequency (3‐5 visits)	0.90 (0.75‐1.08)	0.81* (0.67‐0.98)
High continuity index vs low		
High visit frequency (6 or more visits)	0.79* (0.66‐0.95)	0.94 (0.78‐1.15)
High continuity index vs low		
ACSC admission		
Relational continuity		
Moderate visit frequency (3‐5 visits)	0.83* (0.70‐0.99)	0.83* (0.69‐0.99)
High continuity index vs low		
High visit frequency (6 or more visits)	0.79** (0.66‐0.93)	0.83* (0.69‐0.99)
High continuity index vs low		

Note

Results from model that accounts for unobserved time‐invariant confounding. Continuity: low = ≤median continuity index, high = >median continuity index. Visit frequency: low = 0‐2, moderate = 3‐5, high = 6+ visits in 12 mo. Hazard ratios between two levels of continuity obtained as the ratio of exponentiated coefficients: HR_high/low_ = exp (*β*_high_)/exp (*β*_low_).

* *P* < .05;

** *P* < .01;

*** *P* < .001.

### 3.3 Robustness check results

1. Table S4 shows that varying the minimum number of visits deemed necessary to measure continuity, from three visits in the main analysis to two or four visits, did not substantially change the overall findings.
2. All‐cause unplanned hospital admissions, shown in Table S5, demonstrate a similar pattern to ACSC admissions, with both care plans and higher relational continuity for both moderate‐ and high‐frequency attenders associated with lower hazard of admission.
3. Adding a variable to capture specialist mental health care in the 12‐month lookback period required limiting the observation period to 3 years from April 1, 2011, to March 31, 2014. Results from the shortened observation period are presented with and without the addition of the specialist mental health care variable to allow the impact of each change to be considered separately, as shown in Table S6. The shorter period of observation results in a lack of statistically significant associations between continuity and outcomes, except for a lower risk of ACSC admissions for those with moderate visit frequency. While the specialist mental health care variable is associated with a much higher risk of all three outcomes, its addition does not change the results for the continuity variables.

## 4 DISCUSSION

We found that within‐practice family physician relational continuity for people with SMI was associated with a lower risk of ED presentations and ACSC admissions, and all‐cause unplanned admissions. These effects were present after accounting for time‐invariant confounding, and across three dimensions of relational continuity as captured by three different continuity indices. We did not find significant association between relational continuity and risk of SMI admission. Consistent with a previous study of care plans in family practice for people with SMI,^31^ we found that care plans, which may represent informational/ management continuity, were associated with lower risk of ED presentations, but unlike that study (which did not account for time‐invariant confounding), we found that care plans were also associated with lower risk of SMI admissions. We also found care plans were associated with lower risk of ACSC admissions.

Our results suggest that seeing the same family physician over time can improve the physical health of people with SMI and thereby reduce their need for and use of unplanned hospital care. These findings are consistent with previous studies that found relational continuity to be associated with reduced risk of ACSC admission in a range of different patient groups.^45^, ^52^ Higher continuity of family physician care may reduce the need for hospital care through improved management of physical health, by facilitating familiarity, communication, trust, and quality of relationship between doctor and patient.^15^ The results also suggest that the documentation and sharing of information and management plans across physicians within a family practice can have important benefits for both the physical and mental health of people with SMI. Documentation of care plans was associated with reduced risk of all types of unplanned hospital care.

Our results also highlight the importance of accounting for the individual's propensity to receive continuity of care when studying the impact on outcomes. We used a modeling technique, the correlated random‐effects model, that separates within‐ and between‐individual variation, a method not previously applied (to our knowledge) in the context of continuity of care. The results suggest that unobserved individual factors may drive both the level of continuity of care received, and the risk of unplanned hospital use, and these omitted factors may bias the observed association between continuity and outcomes. The comparison of our main results with those from a model that does not account for this type of endogeneity (the random‐effects model) shows that we would have drawn different conclusions from such an approach. We would have found that care plans were associated with a higher rather than lower risk of SMI admissions, not associated with ACSC admissions, and weakly associated with ED presentations. One explanation for this difference is that people with more severe SMI may be more likely to have a care plan documented and are also more likely to be admitted, which drives the association in the random‐effects model. When we accounted for this unobserved propensity to have a documented care plan overall, having a care plan documented in the prior year was associated with a lower risk of unplanned hospital use. The correlated random‐effects model also showed a weaker association between relational continuity and ED presentations than the random‐effects model, unlike the study by Pu and Chou^35^ which found a stronger effect of continuity when they applied the instrumental variable approach to address endogeneity. However, in addition to the methodological differences, that study looked at across‐practice continuity, which might have different unobserved confounding factors.

In addition to accounting for unobserved time‐invariant factors, other features of our analysis differ from approaches generally taken in this literature. Relational continuity and informational/management continuity (as represented by care plans) were separately captured in the model, which avoided conflating these effects. We also focused on within‐practice continuity, in which different physicians within a practice have access to the same patient records, and may share common approaches to management. We took this approach because within‐practice continuity may be more relevant to family practices than across‐practice continuity, especially in England where patients are registered with a single family practice, and practices can influence which of their doctors see individual patients. Family physician relational continuity in this context may also reflect different factors than in other countries where patients face lower administrative barriers to changing family practice. Where people are free to choose their provider, high relational continuity may reflect a strong, valued therapeutic relationship, which may in turn improve outcomes. In England, people may have more constrained choice of family physician, so that higher relational continuity may be less beneficial.

We found slightly lower levels of continuity than those in an earlier study of family physician continuity for people with long‐term mental illness in the United Kingdom,^53^ but much lower than those found in studies looking only at specialist mental health care.^17^, ^18^ Higher, and rising,^54^ rates of consultation in family practice may contribute to these differences. Relational continuity in English family practices may be affected by reductions in full‐time working and increasing practice size. Average UPC scores for all patients in 2011‐2013 were 0.61.^45^ Comparison with our results suggests this dimension of family physician continuity is not lower for people with SMI than for patients overall.

### 4.1 Limitations

The clinical outcomes we have examined are important as they represent some of the excess health risks for people with SMI, and carry substantial health care costs. However, they are not the only outcomes that matter. Both people with SMI and family physicians value continuity of care in itself as part of how they experience giving and receiving care.^55^, ^56^ Broader outcomes important to people with SMI may also be affected by continuity of care, including social functioning and quality of life.^23^ While our statistical approach accounted for time‐invariant unobserved individual characteristics, we cannot rule out time‐varying confounding that may contribute to our findings. For instance, during periods of deterioration leading to admission, family physicians may have less opportunity to spend time on preventive measures such as care plans. We were unable to differentiate the nature of ED presentations into physical and mental health as done for admissions because this level of detail was not sufficiently recorded in the original data. Care in specialist mental health services might be expected to confound the relationship between continuity in family practice and hospital use. However, we found that although specialist care was strongly associated with higher risk of each outcome, there was no change in the associations between the continuity and each outcome. While this was tested on a smaller sample with a shorter observation period due to data constraints, it provides reassurance that our main results are not biased by the absence of specialist mental health care in the model.

## 5 CONCLUSIONS

Our results suggest that continuity of care in family practice, in terms of relational continuity and information/management continuity, can help to improve both the physical and mental health of people with SMI. Within‐practice relational continuity may reduce the risk of ED presentations and admission to hospital for physical health problems amenable to primary care, and care plans documented by family physicians may reduce the risk of patients presenting to ED or requiring admission. Our findings also suggest that it is important to consider confounding by unobserved individual characteristics when examining the relationship between relational continuity and clinical outcomes. This may be particularly important when considering trade‐offs between continuity of care and other good‐quality aspects of care provision, such as flexibility to respond to urgent needs, or when addressing the resource implications of prioritizing continuity of care in the organization of services.
