# Supplementary material for: Impact of family practice continuity of care on unplanned hospital use for people with serious mental illness
Source: Health Serv Res. 2019 Oct 9;54(6):1316–25. doi: 10.1111/1475-6773.13211 (PMC6863233; doi:10.1111/1475-6773.13211)
Supplement: Supplementary file 2 [file HESR-54-1316-s002.docx]

**Relational continuity of care indices**

$COC=\frac{\left( \sum_{j=1}^{J} n_{j}^{2}-N \right)}{N\left( N-1 \right)}$ (1)

where *N*=total number of visits, and *n_j_*=number of visits with family physician *j*

$UPC= max\left\{ \frac{n_{1}}{N}, \ldots,\frac{n_{j}}{N},\ldots,\frac{n_{J}}{N} \right\}$ (2)

$SECON=\frac{\sum_{i=1}^{N-1} s_{i}}{N-1}$ (3)

where *i* = visit number, *N*=number of visits, *N*-1=number of sequential pairs of visits and

$$s_{i}=\left\{ \begin{aligned} 1 if visit i and visit i+1 are to the same provider \\ 0 \mathrm{otherwise} \end{aligned} \right.$$

**Statistical model**

$h_{it} =Pr \left( T_{i}=t | {T_{i}\geq t;x}_{it};Z_{i};TD;v_{i} \right)$

$=1-exp\{-\exp\left( {X_{it}}^{'}\beta+{+TD^{'}\mu+Z_{i}^{'}\theta}_{i}+v_{i} \right)\}$ (4)

where

$T_{i}$ is the period in which the outcome is observed for individual *i*

*t* represents the time period of interest and defines the risk set consisting of individuals who have not been censored and for whom no outcome has been observed prior to time *t*

*X* are time-varying factors, including a constant term

*TD* is a series of time-period dummy variables representative of the baseline hazard

*Z* are time-invariant factors

$v_{i}\approx N\left( 0,\sigma_{v}^{2} \right)$ is normally distributed individual unobserved heterogeneity

$v_{i}= \alpha+\bar{X}_{i}^{'}\gamma+\bar{TD}^{'}\delta+c_{i}$ (5)

where $\bar{X_{i}}$ is the mean of each time-varying variable and $\bar{TD}$, the mean of the time dummies, $\alpha$ is a constant and $c_{i}$ represents the remaining unobserved heterogeneity assumed to be normally distributed. Equation (5) is inserted into equation (4) which is then estimated as an individual random effects model.

Supplementary Table 1: Read codes used to identify SMI diagnostic categories

| Category | Read codes used to identify diagnostic category |
| --- | --- |
| Schizophrenia and other psychoses | E100.00 E100.11 E100000 E100100 E100200 E100300 E100400 E100500 E100z00 E101.00 E101000 E101400 E101500 E101z00 E102.00 E102000 E102100 E102500 E102z00 E103.00 E103000 E103200 E103300 E103400 E103500 E103z00 E104.00 E105.00 E105000 E105200 E105500 E105z00 E106.00 E107.00 E107.11 E107000 E107100 E107200 E107300 E107400 E107500 E107z00 E10y.00 E10y.11 E10y000 E10y100 E10yz00 E10z.00 E120.00 E121.00 E122.00 E123.00 E123.11 E12y.00 E12y000 E12yz00 E12z.00 E13..00 E13..11 E131.00 E132.00 E133.00 E133.11 E134.00 E13y.00 E13y100 E13yz00 E13z.00 E13z.11 E1z..00 E212200 Eu20.00 Eu20000 Eu20011 Eu20100 Eu20111 Eu20200 Eu20211 Eu20212 Eu20213 Eu20214 Eu20300 Eu20311 Eu20400 Eu20500 Eu20511 Eu20600 Eu20y00 Eu20y12 Eu20y13 Eu20z00 Eu21.00 Eu21.11 Eu21.12 Eu21.13 Eu21.14 Eu21.15 Eu21.16 Eu21.17 Eu21.18 Eu22.00 Eu22000 Eu22011 Eu22012 Eu22013 Eu22014 Eu22015 Eu22100 Eu22111 Eu22200 Eu22300 Eu22y00 Eu22y11 Eu22y12 Eu22y13 Eu22z00 Eu23.00 Eu23000 Eu23011 Eu23012 Eu23100 Eu23112 Eu23200 Eu23211 Eu23212 Eu23214 Eu23300 Eu23312 Eu23y00 Eu23z00 Eu23z11 Eu23z12 Eu24.00 Eu24.12 Eu24.13 Eu25.00 Eu25000 Eu25011 Eu25012 Eu25100 Eu25111 Eu25112 Eu25200 Eu25212 Eu25y00 Eu25z00 Eu25z11 Eu26.00 Eu2y.00 Eu2y.11 Eu2z.00 Eu2z.11 Eu44.14 |
| Bipolar disorder and affective psychoses | E11..00 E11..12 E110.00 E110.11 E110000 E110100 E110200 E110300 E110400 E110600 E110z00 E111.00 E111000 E111100 E111200 E111300 E111400 E111500 E111600 E111z00 E112400 E113400 E114.00 E114.11 E114000 E114100 E114200 E114300 E114400 E114500 E114600 E114z00 E115.00 E115.11 E115000 E115100 E115200 E115300 E115400 E115500 E115600 E115z00 E116.00 E116000 E116100 E116200 E116300 E116400 E116500 E116600 E116z00 E117.00 E117000 E117100 E117200 E117300 E117400 E117500 E117600 E117z00 E11y.00 E11y000 E11y100 E11y300 E11yz00 E11z.00 E11z000 E11zz00 E130.00 E130.11 E13y000 Eu30.00 Eu30.11 Eu30000 Eu30100 Eu30200 Eu30211 Eu30212 Eu30y00 Eu30z00 Eu30z11 Eu31.00 Eu31.11 Eu31.12 Eu31.13 Eu31000 Eu31100 Eu31200 Eu31300 Eu31400 Eu31500 Eu31600 Eu31700 Eu31800 Eu31900 Eu31911 Eu31y00 Eu31y11 Eu31y12 Eu31z00 Eu32300 Eu32311 Eu32312 Eu32313 Eu32314 Eu32800 Eu33213 Eu33300 Eu33311 Eu33312 Eu33313 Eu33314 Eu33315 Eu33316 Eu3z.11 |

Supplementary Table 2: Conditions classified as ambulatory care sensitive admissions^^^

| **Condition** | **ICD-10 codes used to identify the condition**^#^ |
| --- | --- |
| Angina | I10 I24.0 I24.8 I24.9 |
| Asthma | J45 J46 |
| Chronic obstructive pulmonary disease | J41 J42 J43 J44 J47  First diagnosis: J20 + Second diagnosis one of: J41 J42 J43 J44 J47 |
| Congestive heart failure | I50 I11.0 J81 |
| Diabetes (in any diagnosis field) | E10.0 E10.1 E10.2 E10.3 E10.4 E10.5 E10.6 E10.7 E10.8  E11.0 E10.1 E11.2 E10.3 E11.4 E10.5 E11.6 E10.7 E11.8  E12.0 E10.1 E12.2 E10.3 E12.4 E10.5 E12.6 E10.7 E12.8  E13.0 E10.1 E13.2 E10.3 E13.4 E10.5 E13.6 E10.7 E13.8  E14.0 E10.1 E14.2 E10.3 E14.4 E10.5 E14.6 E10.7 E14.8 |
| Epilepsy | G40 G41 O15 R56 |
| Hypertension | I10 I11.9 |
| Anaemia | D50.0 D50.8 D50.9 |
| Cellulitis | L03 L04 L08 L88 L98.0 L98.3 |
| Dehydration | E86 |
| Dental | A69.0 K02 K03 K04 K05 K06 K08 K09.8 K09.9 K12 K13 |
| Ear, nose and throat infections | H66 H67 J02 J03 J06 J31.2 |
| Gangrene (in any diagnosis field) | R02 |
| Gastroenteritis | K52.2 K52.8 K52.9 |
| Nutritional deficiencies | E40 E41 E42 E43 E55 E64.3 |
| Perforated or bleeding ulcer | K25.0 K25.1 K25.2 K25.4 K25.5 K25.6  K26.0 K26.1 K26.2 K26.4 K26.5 K26.6  K27.0 K27.1 K27.2 K27.4 K27.5 K27.6  K28.0 K28.1 K28.2 K28.4 K28.5 K28.6 |
| Urinary tract infection or pyelonephritis | N10 N11 N12 N13.6 N39.0 |
| Influenza (in any diagnosis field, exclude secondary diagnosis of D57) | J10 J11 |
| Pneumonia (in any diagnosis field, exclude secondary diagnosis of D57) | J13 J14 J15.3 J15.4 J15.7 J15.9 J16.8 J18.1 J18.8 |
| Tuberculosis | A15 A16 A19 |
| Other vaccine-preventable diseases (in any diagnosis field) | A35 A36 A37 A80 B05 B06 B16.1 B16.9 B18.0 B18.1 B26 G00.0 M01.4 |

^Based on Bardsley et al. (2013)^41^

#Based on the first diagnosis field in HES data unless otherwise specified.

Supplementary Table 3: Full results for COC index

|  | AE presentation | | SMI admission | | ACSC admission | |
| --- | --- | --- | --- | --- | --- | --- |
|  | HR | *(se)* | HR | *(se)* | HR | *(se)* |
| **Time-varying variables – period level** |  |  |  |  |  |  |
| Continuity/ visit frequency |  |  |  |  |  |  |
| *Base: Low visit frequency, continuity undefined* |  |  |  |  |  |  |
| Moderate frequency, low continuity | 1.07 | *(0.04)* | 1.18* | *(0.08)* | 1.49*** | *(0.13)* |
| Moderate frequency, high continuity | 0.96 | *(0.04)* | 1.16 | *(0.11)* | 1.15 | *(0.10)* |
| High frequency, low continuity | 1.11* | *(0.06)* | 1.55** | *(0.13)* | 2.00*** | *(0.20)* |
| High frequency, high continuity | 1.02 | *(0.05)* | 1.40*** | *(0.14)* | 1.47*** | *(0.15)* |
| Care plan | 0.71*** | *(0.02)* | 0.61*** | *(0.03)* | 0.68*** | *(0.04)* |
| Antipsychotic medication | 0.90* | *(0.04)* | 1.13 | *(0.11)* | 1.12 | *(0.12)* |
| **Time-varying variables – mean level** |  |  |  |  |  |  |
| Continuity/ visit frequency |  |  |  |  |  |  |
| *Base: Low visit frequency, continuity undefined* |  |  |  |  |  |  |
| Moderate frequency, low continuity | 1.56*** | *(0.14)* | 0.61** | *(0.11)* | 1.18 | *(0.12)* |
| Moderate frequency, high continuity | 1.15 | *(0.11)* | 0.51** | *(0.12)* | 0.87 | *(0.20)* |
| High frequency, low continuity | 2.23*** | *(0.18)* | 0.64** | *(0.10)* | 1.45* | *(0.22)* |
| High frequency, high continuity | 1.96*** | *(0.16)* | 0.72 | *(0.12)* | 1.32 | *(0.23)* |
| Care plan | 2.01*** | *(0.15)* | 7.80*** | *(1.00)* | 2.85*** | *(0.41)* |
| Antipsychotic medication | 0.97 | *(0.05)* | 1.06 | *(0.13)* | 0.98 | *(0.13)* |
| **Time-invariant variables (at start of observation)** |  |  |  |  |  |  |
| Index of disadvantage |  |  |  |  |  |  |
| *Base: Quintile 1 – Least disadvantaged* |  |  |  |  |  |  |
| 2 | 1.10* | *(0.05)* | 0.99 | *(0.08)* | 0.93 | *(0.06)* |
| 3 | 1.18*** | *(0.05)* | 1.03 | *(0.08)* | 1.08 | *(0.07)* |
| 4 | 1.26*** | *(0.06)* | 1.00 | *(0.08)* | 1.20** | *(0.09)* |
| Quintile 5 - Most disadvantaged | 1.44*** | *(0.08)* | 1.10 | *(0.10)* | 1.28** | *(0.10)* |
| Ethnicity |  |  |  |  |  |  |
| *Base: black & minority ethnicities* |  |  |  |  |  |  |
| White | 1.49*** | *(0.04)* | 1.80*** | *(0.14)* | 2.11*** | *(0.16)* |
| History of smoking |  |  |  |  |  |  |
| *Base: non-smoker* |  |  |  |  |  |  |
| Current or ex-smoker | 1.07* | *(0.03)* | 0.96 | *(0.05)* | 1.03 | *(0.06)* |
| Age |  |  |  |  |  |  |
| *Base: 18-35* |  |  |  |  |  |  |
| 36-45 | 0.85*** | *(0.03)* | 1.01 | *(0.06)* | 1.11 | *(0.09)* |
| 46-55 | 0.78*** | *(0.03)* | 0.76*** | *(0.05)* | 1.46*** | *(0.12)* |
| 56-65 | 0.83*** | *(0.03)* | 0.66*** | *(0.04)* | 2.11*** | *(0.17)* |
| >=66 | 1.18*** | *(0.04)* | 0.49*** | *(0.05)* | 3.88*** | *(0.35)* |
| Years since SMI diagnosis |  |  |  |  |  |  |
| *Base: 0-1 year* |  |  |  |  |  |  |
| 2-5 | 1.04 | *(0.03)* | 1.08 | *(0.07)* | 1.21* | *(0.10)* |
| >5 | 0.94* | *(0.03)* | 1.09 | *(0.07)* | 1.22** | *(0.08)* |
| Sex |  |  |  |  |  |  |
| *Base: female* |  |  |  |  |  |  |
| Male | 1.04 | *(0.02)* | 1.00 | *(0.04)* | 1.08 | *(0.05)* |
| SMI diagnosis category |  |  |  |  |  |  |
| *Base: bipolar disorder, affective psychosis* |  |  |  |  |  |  |
| Schizophrenia or other psychosis | 0.97 | *(0.03)* | 1.24*** | *(0.07)* | 1.04 | *(0.06)* |
| Both categories | 0.99 | *(0.03)* | 1.99*** | *(0.13)* | 0.96 | *(0.07)* |
| Comorbidity |  |  |  |  |  |  |
| Number of Charlson comorbidities | 1.11*** | *(0.02)* | 0.92* | *(0.04)* | 1.38*** | *(0.04* |
| Comorbid depression | 1.02 | *(0.02)* | 0.79*** | *(0.04)* | 1.05 | *(0.05)* |
| Observations | 203,534 |  | 281,017 |  | 286,940 |  |
| Number of individuals | 19,324 |  | 19,324 |  | 19,324 |  |

Cluster-robust standard errors in parentheses

*** p<0.001, ** p<0.01, * p<0.05

HR: Hazard ratio (exponentiated coefficient)

Continuity: low= ≤ median COC index, high= > median COC index

Visit frequency: low=0-2, moderate=3-5, high=6+ visits in 12 months

Supplementary Table 4. Key variables, varying the minimum number of visits for the measurement of COC index

|  | 3 visits (main) | 2 visits | 4 visits |
| --- | --- | --- | --- |
|  | Hazard ratio (95%CI) | Hazard ratio (95%CI) | Hazard ratio (95%CI) |
| ED presentation |  |  |  |
| *Relational continuity* |  |  |  |
| Moderate visit frequency (3-5 visits)  High COC index vs. low COC index | 0.89**  (0.83-0.96) | 0.90**  (0.84-0.96) | 0.86**  (0.78-0.95) |
| High visit frequency (6 or more visits)  High COC index vs. low COC index | 0.92  (0.84-1.00) | 0.92  (0.84-1.00) | 0.92  (0.84-1.00) |
| SMI admission |  |  |  |
| *Relational continuity* |  |  |  |
| Moderate visit frequency (3-5 visits)  High COC index vs. low COC index | 0.98  (0.82-1.18) | 0.90  (0.77-1.04) | 0.89  (0.72-1.10) |
| High visit frequency (6 or more visits)  High COC index vs. low COC index | 0.90  (0.75-1.08) | 0.89  (0.74-1.07) | 0.90  (0.75-1.08) |
| ACSC admission |  |  |  |
| *Relational continuity* |  |  |  |
| Moderate visit frequency (3-5 visits)  High COC index vs. low COC index | 0.77**  (0.65-0.91) | 0.79**  (0.67-0.92) | 0.76*  (0.61-0.95) |
| High visit frequency (6 or more visits)  High COC index vs. low COC index | 0.73***  (0.62-0.87) | 0.73***  (0.62-0.87) | 0.74***  (0.62-0.88) |

*p<0.05, **p<0.01, ***p<0.001

Continuity: low= ≤ median COC index, high= > median COC index

Visit frequency: low=0-minimum, moderate=minimum-5, high=6+ visits in 12 months

Hazard ratios between two levels of continuity obtained as the ratio of exponentiated coefficients: ${HR}_{high/low}=\exp\left( \beta_{high} \right)/exp\left( \beta_{low} \right)$

Supplementary Table 5. Association between continuity measures and outcome of any unplanned hospital admission (physical or mental health conditions)^

|  | Hazard ratio (95%CI) |
| --- | --- |
| Any unplanned admission |  |
| *Relational continuity* |  |
| Moderate visit frequency (3-5 visits)  High COC index vs. low COC index | 0.85**  (0.76-0.94) |
| High visit frequency (6 or more visits)  High COC index vs. low COC index | 0.86**  (0.78-0.95) |
| *Information/ management continuity* |  |
| Care plan vs. none | 0.67***  (0.62-0.72) |

*p<0.05 **p<0.01 ***p<0.001

^Results from a correlated random effects model, with 3 visits as the minimum level for measuring COC index in the 12-month lookback period

Visit frequency: low=0-2, moderate=3-5, high=6+ visits in 12 months

Hazard ratios between two levels of continuity obtained as the ratio of exponentiated coefficients: ${HR}_{high/low}=\exp\left( \beta_{high} \right)/exp\left( \beta_{low} \right)$

Supplementary Table 6. Association between continuity measures and outcomes in observation period 2011-2014, and with additional covariate of treatment in specialist mental health services (N=15,364)

|  | Observation 2011-2014 | Plus specialist mental health care |
| --- | --- | --- |
|  | Hazard ratio (95%CI) | Hazard ratio (95%CI) |
| ED presentation |  |  |
| *Relational continuity* |  |  |
| Moderate visit frequency (3-5 visits)  High COC index vs. low COC index | 0.93  (0.84-1.04) | 0.93  (0.84-1.04) |
| High visit frequency (6 or more visits)  High COC index vs. low COC index | 0.95  (0.85-1.08) | 0.95  (0.85-1.07) |
| *Informational/ management continuity* |  |  |
| Care plan vs. none | 1.01  (0.93-1.11) | 1.00  (0.92-1.09) |
| *Specialist mental health care* |  |  |
| Any vs. none |  | 1.78***  (1.58-2.01) |
| SMI admission |  |  |
| *Relational continuity* |  |  |
| Moderate visit frequency (3-5 visits)  High COC index vs. low COC index | 1.16  (0.11-11.83) | 1.13  (0.86-1.49) |
| High visit frequency (6 or more visits)  High COC index vs. low COC index | 0.88  (0.33-2.39) | 0.90  (0.68-1.17) |
| *Informational/ management continuity* |  |  |
| Care plan vs. none | 0.93  (0.03-25.16) | 0.94  (0.78-1.14) |
| *Specialist mental health care* |  |  |
| Any vs. none |  | 3.79***  (2.90-4.94) |
| ACSC admission |  |  |
| *Relational continuity* |  |  |
| Moderate visit frequency (3-5 visits)  High COC index vs. low COC index | 0.74*  (0.55-0.98) | 0.73*  (0.55-0.98) |
| High visit frequency (6 or more visits)  High COC index vs. low COC index | 0.78  (0.60-1.02) | 0.78  (0.60-1.03) |
| *Informational/ management continuity* |  |  |
| Care plan vs. none | 1.17  (0.98-1.39) | 1.15  (0.96-1.36) |
| *Specialist mental health care* |  |  |
| Any vs. none |  | 2.38***  (1.88-3.01) |

*p<0.05 **p<0.01 ***p<0.001

^Results from a correlated random effects model, with 3 visits as the minimum level for measuring COC index in the 12-month lookback period

Visit frequency: low=0-2, moderate=3-5, high=6+ visits in 12 months

Hazard ratios between two levels of continuity obtained as the ratio of exponentiated coefficients: ${HR}_{high/low}=\exp\left( \beta_{high} \right)/exp\left( \beta_{low} \right)$

Results from the shortened observation period are presented with and without the addition of the specialist mental health care variable to allow the impact of each change to be considered separately.
